# Interleukin-1-related activity and hypocretin-1 in cerebrospinal fluid contribute to fatigue in primary Sjögren’s syndrome

**DOI:** 10.1186/s12974-019-1502-8

**Published:** 2019-05-17

**Authors:** Kjetil Bårdsen, Cato Brede, Ingeborg Kvivik, Jan Terje Kvaløy, Kristin Jonsdottir, Anne Bolette Tjensvoll, Peter Ruoff, Roald Omdal

**Affiliations:** 1 Research Department, Stavanger University Hospital, Stavanger, Norway; 2 Department of Chemistry, Bioscience and Environmental Engineering, University of Stavanger, Stavanger, Norway; 3 Department of Medical Biochemistry, Stavanger University Hospital, Stavanger, Norway; 4 Department of Mathematics and Physics, University of Stavanger, Stavanger, Norway; 5 Department of Neurology, Stavanger University Hospital, Stavanger, Norway; 6 Centre for Organelle Research (CORE), Faculty of Science and Technology, University of Stavanger, Stavanger, Norway; 7 Clinical Immunology Unit, Department of Internal Medicine, Stavanger University Hospital, POB 8100, N-4068 Stavanger, Norway; 8 Department of Clinical Science, Faculty of Medicine, University of Bergen, Bergen, Norway

**Keywords:** Innate immunity, Cytokines, Sjögren’s syndrome, Fatigue, Hypocretin

## Abstract

**Background:**

Fatigue is a common and sometimes debilitating phenomenon in primary Sjögren’s syndrome (pSS) and other chronic inflammatory diseases. We aimed to investigate how IL-1 β-related molecules and the neuropeptide hypocretin-1 (Hcrt1), a regulator of wakefulness, influence fatigue.

**Methods:**

Hcrt1 was measured by radioimmunoassay (RIA) in cerebrospinal fluid (CSF) from 49 patients with pSS. Interleukin-1 receptor antagonist (IL-1Ra), IL-1 receptor type 2 (IL-1RII), IL-6, and S100B protein were measured by enzyme-linked immunosorbent assay (ELISA). Fatigue was rated by the fatigue visual analog scale (fVAS).

**Results:**

Simple univariate regression and multiple regression analyses with fatigue as a dependent variable revealed that depression, pain, and the biochemical variable IL-1Ra had a significant association with fatigue. In PCA, two significant components were revealed. The first component (PC1) was dominated by variables related to IL-1β activity (IL-1Ra, IL-1RII, and S100B). PC2 showed a negative association between IL-6 and Hcrt1. fVAS was then introduced as an additional variable. This new model demonstrated that fatigue had a higher association with the IL-1β-related PC1 than to PC2. Additionally, a third component (PC3) became significant between low Hcrt1 concentrations and fVAS scores.

**Conclusions:**

The main findings of this study indicate a functional network in which several IL-1β-related molecules in CSF influence fatigue in addition to the classical clinical factors of depression and pain. The neuropeptide Hcrt1 seems to participate in fatigue generation, but likely not through the IL-1 pathway.

**Electronic supplementary material:**

The online version of this article (10.1186/s12974-019-1502-8) contains supplementary material, which is available to authorized users.

## Background

Fatigue can be defined as “an overwhelming sense of tiredness, lack of energy, and feeling of exhaustion” [1] and is a common phenomenon in infections, chronic inflammatory diseases, cancer, and neurodegeneration. Fatigue has a substantial impact on patients’ lives, is sometimes debilitating, and is a major reason for using sick leave. It remains unclear whether there are different dimensions of fatigue, such as peripheral (muscle) and central (mental) fatigue, or whether fatigue is a unidimensional phenomenon that influences different aspects of human life.

While the underlying mechanisms are not completely understood, several studies have shown that pain and depression are factors that exert a heavy and consistent influence on the severity of fatigue [2, 3]. Most researchers regard fatigue as a biological and cerebral phenomenon, and increasing evidence points to a genetic and molecular basis for the generation and regulation of fatigue [4, 5].

A conceptual model for understanding fatigue is the *sickness behavior* phenomenon in animals [6, 7]. “Sickness behavior” is observed during infection and inflammation and is characterized by sleepiness, depressive mood, social withdrawal, and loss of grooming, thirst, appetite, and initiative [8]. Sickness behavior can be considered as a survival-enhancing strategy and is highly conserved during evolution [9]. Fatigue constitutes a substantial part of this behavior.

Several animal studies have explored the pathways involved in sickness behavior and demonstrated the fundamental role of interleukin (IL)-1β signaling to the brain for this complex and automated response [6]. In conditions with infection and/or tissue injury, activation of innate immunity cells such as macrophages will rapidly lead to increased production of IL-1β. This activates other immune cells to destroy and eliminate the pathogen or the endogenous danger molecules. In the periphery, IL-1β signals through binding the IL-1 receptor type 1 (IL-1RI) and the IL-1R accessory protein (IL-1RAcP) [7]. Activation of this signaling complex gives rise to the canonical downstream IL-1 responses (NF-κB and MAPKs) with increased inflammation and immune cell activation. Downregulation of IL-1 induced receptor activation is conducted by the IL-1 receptor type II (IL-1RII), which functions as a decoy receptor and does not signal, and by the natural IL-1 receptor antagonist (IL-1Ra), which binds to the IL-1RI and prevents activation [10]. Without these limiting steps, IL-1-driven inflammation can run rampant.

IL-1β passes through the blood-brain barrier (BBB) and reaches neuronal cells in the brain by both passive and active transport systems and can even be produced intrathecally [11]. Once in the brain, IL-1β binds to a subtype of the IL-1 receptor and to a brain isoform of the accessory protein, the IL-1RaAcPb [12]. Thus, while IL-1β in the periphery is a strong inducer of innate immunity-based inflammation, IL-1β directly modulates synaptic transmission through neuronal potassium and calcium influx (without inflammation) in the brain [12, 13] and induces sickness behavior. IL-1Ra is a robust biomarker in CSF and is in equilibrium with IL-1β in chronic conditions. High levels of IL-1Ra in CSF therefore are thought to reflect high IL-1β levels.

In humans, increased activation of IL-1 in the brain is observed in chronic inflammatory and autoimmune conditions [14, 15], and treatment with IL-1 blocking agents alleviates fatigue [16–18]. In primary Sjögren’s syndrome (pSS), a chronic autoimmune disease clinically characterized by inflammation of the exocrine glands, fatigue is a dominant feature [19]. No consistently effective treatments are currently available, and pSS can be considered as an ideal disease to investigate fatigue mechanisms as gene expression and molecular pathways at large are undisturbed by immunosuppressive or cytotoxic drug treatment. To further explore models for fatigue regulation, we therefore performed measurements of selected molecules in cerebrospinal fluid (CSF) that could influence Il-1β activity (IL-1Ra, IL-1RII, IL-6, and S100B).

Animal studies show that lipopolysaccharide (LPS)-induced sickness behavior also is accompanied by reduced *c-fos* activity of lateral hypothalamic neurons that produce hypocretin-1 (Hcrt1) and also a reduction of Hrct1 levels in CSF [6, 20]. Since Hcrt1 is known as the main regulator of sleep and wakefulness, we also hypothesize that this neuropeptide could be another molecular regulator of fatigue.

## Methods

This hypothesis-generating study was designed to explore associations between the CSF concentrations of selected molecules and fatigue in a population of patients with pSS at a single institution.

All patients with systemic autoimmune diseases in the Southern part of Rogaland County are allocated to Stavanger University Hospital. We reviewed medical records and identified 99 patients that fulfilled the American-European Consensus Group (AECG) criteria for pSS [21]. None of the patients were on biological drug treatment. Exclusion criteria were past head and neck radiation treatment, hepatitis C infection, acquired immunodeficiency syndrome (AIDS), pre-existing lymphoma, sarcoidosis, graft versus host disease, and use of anticholinergic drugs [21]. Seventy-two patients consented to participate in the study and subjected to a 2-day stay in the hospital for research purposes only. The patients in the near population-based cohort were on no biological drug treatment. Fifty-five of the 72 patients (76%) consented to lumbar puncture. One of the 55 patients was later excluded due to a brain tumor revealed by MRI, three because of blood contamination of the CSF, and another two because of inadequate sample volumes. Thus, CSF samples from 49 out of the 72 pSS patients (68%) were available for study, 41 women (84%) and 8 men (16%).

Blood was drawn in the morning between 08.00 and 09.00 a.m, and clinical examinations and patient-reported outcome measures completed afterwards. Lumbar puncture was performed between 01:00 and 02:00 pm; samples were collected in cooled glass tubes and immediately placed on ice until centrifugation at 3000 *g* for 10 min at 4 °C. The supernatants were distributed in 200 μL aliquots and stored at − 70 °C until analysis. CSF samples from three patients were excluded because of blood contamination, and another two because of inadequate sample volumes. Thus, CSF samples from 50 pSS patients were available for study. In addition to clinical variables given in Table 1, four patients (8 %) had a BMI ≥ 30, 18 patients (36 %) were on antimalarial drugs, and the median duration of education was 12.3 years (range 7–20 years).

**Table 1:** Selected clinical variables in the 49 pSS patients

Variables	
Females (%)/males (%)	41 (84)/8 (16)
Age, years, median [range]	56.1 [34.2–78.2]
Duration, years, median [range]	5.0 [0.4–16.0]
fVAS scores, median [range]	64 [3.0–93.0]
BDI scores, median [range]	9.0 [0.0–38.0]
Pain scores*, median [range]	49.0 [0.0–90.0]
Presence of anti-SSA/SSB antibodies (%)	38 (78)
CRP (mg/L) [range]	1.5 [0.0–9.0]

*Abbreviations*: *fVAS* fatigue visual analog scale, *BDI* Beck Depression Inventory, *SSA/SSB* Sjögren’s syndrome-related antigen A (Ro) and B (La), *CRP* C-reactive protein

*SF-36 pain scores are reported according to the transformed scale with high scores indicating high bodily pain and low scores indicating lower bodily pain

Fatigue was assessed by the fatigue visual analog scale (fVAS), which is a generic and unidimensional fatigue instrument that has been widely used to measure fatigue in patients with various conditions [22]. It consists of a 10- mm horizontal line with vertical anchoring lines. The description at the left end (0 mm) is “no fatigue,” and the description at the right end (100 mm) is “fatigue as bad as it can be.” The subjects are asked to draw a vertical line at the point corresponding to their experience of fatigue the last week, and the distance from the left anchor is measured, yielding a numerical score for fatigue (Additional file 1: Figure S1).

Mood was assessed by the Beck Depression Inventory (BDI) [23]. A BDI score of < 13 is normally regarded as no depression; a score of 13–19 represents mild depression and a score of >19 reflects moderate-to-severe depression.

Pain was assessed by the pain subscale of the Medical Outcome Survey (MOS) short form-36 (SF-36) questionnaire and transformed as recommended [24].

For regression analysis and principal component analysis (PCA), the transformed pain scale was inverted by subtracting the transformed score from 100. This was performed to orient the pain scale in the same direction as scales for other variables included in the analyses.

IL-1Ra and IL-6 were analyzed on a Luminex^100^ instrument (Luminex Corp., Austin, TX). IL-1Ra was measured using a Fluorokine MAP human IL-1Ra kit (R&D Systems, Minneapolis, MN) and a Fluorokine MAP human base kit (R&D Systems) according to manufacturer’s protocols. Intra-assay CV% was 2.8–4.4, and inter-assay CV% was 6.6–10.9. IL-6 was measured using a human IL-6 ultrasensitive AB bead kit with the human extracellular buffer kit (Biosource, Invitrogen Corp., Carlsbad, CA). Intra-assay CV% was 7.59 and inter-assay CV% was 9.9. For both IL-1Ra and IL-6, acquired data were studied using the StarStation software v2.3 (Applied Cytometry, Sheffield, UK). In the IL-6 assay, nine samples had values below the standard curve and were given the value of the detection limit (1.0 pg/mL) divided by the square root of 2.

Because IL-1β is difficult to measure in CSF due to low concentrations, these results were not included.

S100B and IL-1RII concentrations were measured by sandwich ELISA kits according to the manufacturer’s protocol (S100B: Abnova, Taipei City, Taiwan; IL-1RII: R&D Systems, Minneapolis, MN). ELISA plates were read on a Multiskan Ascent microplate reader (Thermo Scientific, Waltham, MA). For S100B ELISA kits, the intra-assay and inter-assay CV% were 1.9–2.1 and 4.7–7.1, respectively. For IL-1RII, the intra-assay CV% was 2.0–3.4 and the inter-assay CV% was 3.9–5.9.

The concentration of Hcrt1 in CSF was measured by I^125^ radioimmunoassay (RIA) (Phoenix Pharmaceuticals, Burlingame, CA, USA) per manufacturer’s protocol. Samples were measured in duplicate and assay tubes were counted on a RIASTAR gamma counter (Perkin Elmer, USA). Briefly, 100 μL of standard dilution, assay controls, and CSF samples were added to assay tubes together with 100 μL primary antibody and incubated for 20 h at 4 °C before adding 100 μL of ^125^I-peptide (tracer solution) and a new incubation for 20 h at 4 °C. After the second incubation, 100 μL of goat anti-rabbit serum and 100 μL normal goat serum was added. Following incubation for 90 min at room temperature, the assay tubes were centrifuged at 300 rpm for 20 min at 4 °C and incubated for 90 min at room temperature. The supernatant was aspirated before counts per million was counted from the remaining pellet. The average CSF Hcrt1 concentration was 239.3 ± 26.8 pg/mL. Intra-assay variation was 9.9% based on a sample assayed as ten individual samples.

### Statistics

Some clinical variables were not normally distributed, and all continuous data are therefore reported as medians and ranges. Categorical data are reported as counts and percentages. Simple univariate linear regression was first used to examine associations between fatigue and each of the potential influential factors, BDI, pain scores, IL-1Ra, IL-1RII, IL-6, S100B, and Hcrt1. Thereafter, a multiple regression analysis with forward and backward selection was performed to investigate the mutual effect of these factors on fatigue. Variables selection in the final model was based on variables with a significant contribution (*p* < 0.05) and the lowest Akaike’s information criterion (AIC) value. AIC evaluates the multiple regression model by favorizing higher explained variance and penalizing the number of variables in the model. Thus, the model with the lowest AIC was selected.

To further explore and visualize the complex interaction of multiple clinical and laboratory variables on fatigue, we applied principal component analysis (PCA). Centering and standardizing of the data were performed before analysis to avoid effects due to differences in units of the variables. The components retained in PCA were those with eigenvalues > 1. Score distance plots and orthogonal distance plots were used to detect possible outliers in PCA. Samples outside a critical boundary, the 97.5 % quantile in these plots, were declared as outliers.

All analyses were performed in R version 3.3.3 using RStudio version 1.0.144. PCA was performed using the R package FactoMinerR.

## Results

Patient characteristics are summarized in Table 1. There was no significant difference in routine hematological or biochemical variables between patients with high or low fatigue. Diagnostic evaluation of the PCA revealed that one sample was classified as an orthogonal outlier. This sample was removed and PCA was thus performed on 48 samples.

The clinical variables of depression and pain and the biochemical variable IL-1Ra were significantly associated with fatigue in simple univariate linear regression, while no significant associations were observed for Il-1RII, IL-6, S100B, or Hcrt1 (Table 2). In multiple linear regression analysis with fVAS as the dependent variable, both forward and backward stepwise selection resulted in a model with depression, pain, and IL-1Ra as significant independent variables (*R*^2^ = 0.37; *p* < 0.001, Table 3). To obtain an unsupervised impression of the complex molecular interactions, we first performed PCA on a model containing only the biochemical variables. Data from the biochemical variables were centered and scaled before analysis and PCA was performed on the correlation matrix. Two components demonstrated eigenvalues > 1 in a Scree plot and were retained (Additional file 2: Figure S2a). These two components explained 62.77 % of the variation in the dataset.

**Table 2:** Associations (simple regression analysis) between fatigue (fVAS scores) and selected variables

Independent variables	Estimate	Std. error	*R* ^2^	*P*
Depression (BDI) scores	1.42	0.42	0.20	0.001
Pain (SF-36) scores*	0.48	0.13	0.23	< 0.001
IL-1Ra	0.52	0.24	0.09	0.04
IL-1RII	0.13	0.16	0.01	0.41
IL-6	− 3.47	2.43	0.04	0.16
S100B	0.05	0.04	0.03	0.22
Hcrt1	− 0.04	0.14	0.002	0.76

*Abbreviations*: *BDI* Beck Depression Inventory, *SF-36* 36-item short form survey instrument, *IL-1Ra* interleukin 1 receptor antagonist, *IL-1RII* interleukin 1 receptor type 2, *IL-6* interleukin 6, *S100B* S100 calcium binding-protein B, *Hcrt1* hypocretin 1

*SF-36 pain scores are reported according to the transformed scale with high scores indicating high bodily pain and low scores indicating low bodily pain

**Table 3:** Multiple regression model of fatigue (fVAS scores) and selected variables

Variables	Estimate	Std. error	*P*
Depression (BDI) scores	0.97	0.40	0.019
Pain (SF-36) scores*	0.38	0.13	0.004
IL-1Ra	0.54	0.19	0.009

Statistics for the final model: adjusted *R*^2^ 0.37, *P* value < 0.001

*Abbreviations*: *BDI* Beck Depression Inventory, *SF-36* 36-item short form survey instrument, *IL-1Ra* interleukin 1 receptor antagonist

*SF-36 pain scores are reported according to the transformed scale with high scores indicating high bodily pain and low scores indicating low bodily pain

**Fig. 1:**
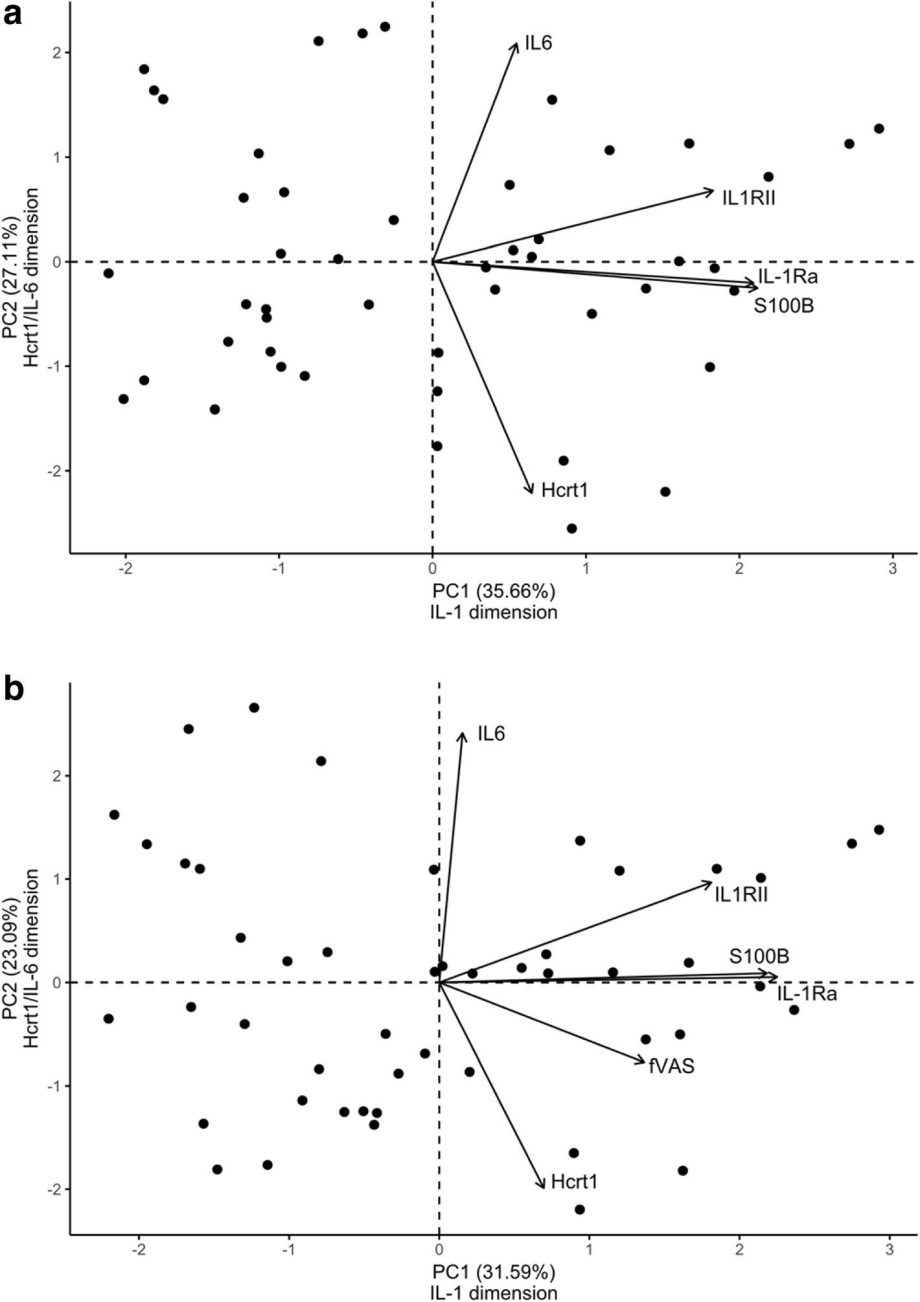
**a** PCA of the biochemical variables only. **b** PCA of the biochemical variables and fatigue variable (fVAS). Bi-plot shows scores of the individuals and variable correlations for PC1 and PC2. Individual scores are illustrated by dots. Arrows illustrate the correlations of the variables to the components. Longer arrows mean higher correlation and arrows close to a component has a higher contribution in the generation of the component

In the PCA bi-plot (Fig. 1a, b), patients are illustrated as dots and the variables as arrows. The length of an arrow is a function of its magnitude, and variables with longer arrows in the direction of a principal component will contribute most to the generation of this specific component.

The first component (PC1) explained most of the variance (35.66 %) (Fig. 1a), and the variable with the highest correlation with the first dimension was S100B (0.79), followed by IL-1Ra (0.78) and IL-1RII (0.68). The fatigue-related variable IL-1Ra revealed in simple and multiple regression analyses was thus highly correlated with S100B and IL-1RII. These results indicate that the first dimension was dominated by variables related to IL-1β activity, *the IL-1 dimension*.

The second component (PC2)—*the Hcrt1/IL-6 dimension*—explained 27.11 % of the variance in the dataset (Fig. 1a). Hcrt1 and IL-6 were the variables with the highest correlation, 0.82 and 0.78, respectively. The negative correlation of Hcrt1 in the second dimension indicates that individuals with low CSF concentrations of Hcrt1 also had high IL-6 concentrations.

In a second PCA model (Fig. 1b), fVAS scores were introduced as an additional variable to explore how fatigue contributed to the data cloud together with the biochemical components. In this model, PCA resulted in three components with eigenvalues > 1 explaining 71.63 % of the variance in the dataset (Additional file 2: Figure S2b). IL-1Ra showed the highest correlation (0.79) with PC1, followed by S100B (0.76), IL-1RII (0.63), and fVAS (0.48). IL-6 had the highest correlation (0.84) with PC2, followed by a negative Hcrt1 correlation (− 0.70). In the third component (data not shown), the variables with the highest correlation were fatigue (− 0.76) and Hcrt1 (0.58).

Adding fatigue-scores to the PCA resulted in a new data cloud in which the composition of the two first components was similar to the components in the PCA model without fatigue (Fig. 1a). Thus, rather than generating new dimensions or changing the composition of the components in the data cloud, fatigue showed a moderate association with variables on PC1 and a strong negative association with Hcrt1 on PC3.

## Discussion

The main findings in this study indicate a complex functional network in which clinical phenomena (pain and depression) together with several IL-1-related molecules in CSF influence fatigue in the context of sickness behavior. In addition, a role for the neuropeptide Hcrt1 as a fatigue-inducing molecule emerges.

**Fig. 2:**
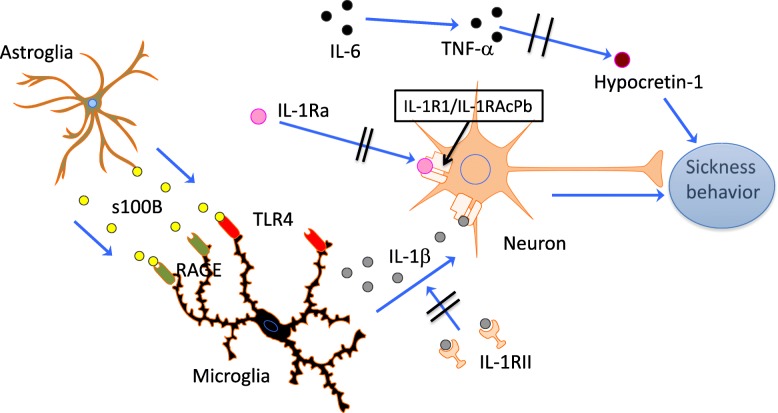
Hypothetical model for fatigue. Danger signals activate astroglia to produce and release S100B that bind to TLR4 and RAGE receptors on microglia. Activated microglia produce IL-1β that bind specific receptors (IL-1R1/IL-1RAcPb) on neurons and induce sickness behavior. There are no specific receptors for IL-6 that can modulate neurons in the same manner as for IL-1β, but higher levels of IL-6 may through stimulation of TNFα synthesis reduce the amount of Hcrt-1 in neurons. Low levels of Hcrt-1 can lead to or modulate fatigue through unknown pathways

Regarding molecules that relate to the IL-1 network, it is difficult to note the specific actions and relative importance of different molecules, but a common denominator seems to be a final IL-1β signaling of fatigue in the brain. The influence of the other biomarkers measured in CSF “disappear” in the complex biological network interactions and only become evident in more advanced statistical models. In addition, the neuropeptide Hrct1—the main regulator of sleep and wakefulness—could represent a parallel, alternative, or redundant fatigue mechanism operative in inflammatory conditions and possibly driven by IL-6/TNF-α modulation of Hcrt1 (Fig. 2).

As shown in a number of previous studies, the clinical factors of pain and depression have a strong influence on fatigue. Whether these factors modulate the fatigue experience on a more psychological basis, whether pain generates fatigue through neuropeptide signaling, or if fatigue and depression are both signaled by IL-1 is not understood. Both animal and human studies have documented IL-1 signaling as one of the mechanisms underlying depression [25, 26]. Depression is also an important component of the sickness behavior response, in which fatigue is such a dominant phenomenon. The close association between fatigue and depression observed in so many studies may therefore have a biological explanation.

Pain is also a well-known associate of fatigue; many different explanations have been proposed to explain this constellation. Patients with chronic pain syndromes such as fibromyalgia consistently report fatigue. Recently, we revealed that fatigue in patients with pSS was associated with variance in a gene coding for an opioid transporter protein in which the more common allele had more severe fatigue [27]. One could therefore hypothesize that pain in an evolutionary perspective is a “danger signal” that induces sickness behavior. Regarding depression, pain and fatigue may therefore also have a biological basis.

In regard to biochemical factors, animal and human studies in acute and chronic inflammation show that IL-1 signaling is crucial for the sickness behavior response, in which fatigue is a major element. Most human studies using peripheral blood have not been able to demonstrate an influence of any cytokine on fatigue. Peripherally produced cytokines and other molecules have to cross the blood-brain barrier to act on the brain, where fatigue is generated. However, cytokines act in complex networks and the effect of one single cytokine upon a dependent factor such as fatigue can be difficult to evaluate. In the CSF, this can be even more problematic, as, for example, IL-1β appears in very low concentrations. Traditional statistical approaches may not detect effects unless they are extremely dominating. To decipher complex interactions in systems-biology research, it may therefore be of benefit to use alternative statistical approaches such as PCA. In this study, the initial regression analyses only found IL-1Ra of the biochemical factors to be a significant contributor, while in the PCA analyses, a logical pattern of molecular interactions for fatigue regulation became evident. A caveat in the deduction of processes that drive PCA dimensions is that other unmeasured covariates could be operative. It is for example known that IL-6 can elicit an IL-1Ra response independent of IL-1β secretion [28]. Nevertheless, these observations expand the knowledge of how fatigue can be generated and allows a more comprehensive understanding of the biological basis of fatigue in inflammatory and possibly also in non-inflammatory conditions.

In the proposed model for fatigue (Fig. 2), IL-1β can either originate from the periphery after passing the blood-brain barrier or be produced intrathecally by activated microglia. In the latter case, protein S100B secreted by activated astrocytes can signal through RAGE and TLR4 on microglia and lead to IL-1β production [29, 30]. Astrocytes are the most numerous cells in the mammalian brain and have a wide range of functions. They comprise an important component of the blood-brain barrier and contact endothelial cells with their end-feet. The importance of astrocytes in innate and adaptive immune responses in the brain has become more clear in recent years [31]. Activation of astrocytes and microglia may therefore in states of inflammation be the initial step in fatigue generation and could possibly also represent a mechanism by which states with cellular stress or “danger” such as degenerative diseases and cancer induce fatigue through microglia IL-1β production.

Sickness behavior is regarded as an important survival factor in evolution, and it is therefore plausible that pathways other than IL-1-driven mechanisms may have developed and can be operative in non-inflammatory and malignant conditions. In this context, Hcrt1 is an interesting candidate. Lack of Hcrt1 is the cause of narcolepsy type 1 [32, 33], and chronic fatigue is prevalent and strong in this condition [34]. It is known that low levels of Hcrt1 also lead to reduced appetite [35]. Low levels of Hcrt1 in CSF may therefore influence both appetite and fatigue, two prominent components of the sickness behavior response.

Studies on Hrct1 and fatigue are rare. There are several case reports of multiple sclerosis patients with hypothalamic lesions with low Hrct1 in CSF and with accompanying hypersomnia or fatigue, but cohort studies are conflicting; for a review, see Burfeind et al. [36]. In cancer, fatigue is common and worsens during cytotoxic treatment. One study in rodents showed that suppression of activity in the hypothalamic Hcrt-producing neurons and low Hcrt1 concentrations in CSF occurred during cytotoxic treatment and had a causal role in chemically induced fatigue [37]. When administered to humans, hypocretin receptor antagonists typically are associated with the side effects of sleepiness and fatigue, indicating that when hypocretin receptors are blocked, subjects experience fatigue [38].

In our study, high inflammatory activity in CSF (IL-6 high) was accompanied by low Hcrt1 (Fig. 1a, b). IL-6 that can be produced by a variety of cells induces production of TNF-α, which again is able to downregulate mRNA prepro-hypocretin, the precursor of Hrct1 [39]. Unfortunately, we did not measure TNF-α in this study and can therefore only speculate that in states of infection, damage or immunological danger, IL-6 leads to low levels of Hcrt1 and induces fatigue via a TNF-α-dependent mechanism. Although the target receptor or cells are unknown, this pathway may represent an alternative fatigue mechanism besides IL-1 signaling.

Limitations of this study include the small number of patients in the cohort and the low number of variables included in the analysis. A larger cohort would provide higher statistical power for both univariate and multiple regression analyses and the PCA. TNF-α analyses in CSF would have provided a greater impact on the Hcrt1 hypothesis for fatigue signaling. Measures of IL-β would have provided a better understanding of its role in the proposed network. Due to the very low CSF concentrations, robust data is difficult to obtain. However, since IL-1Ra downregulates IL-1β activity, it can be considered as a surrogate marker for IL-1β. Strong arguments in support of this is a study of patients with aseptic meningitis revealing CSF IL-1Ra levels approximately 2,000 times the level of IL-1β. IL-1Ra levels peaked about 12 h after IL-1β levels [40]. Another study in animals found that hypothalamic IL-1β mRNA peaked 1 h after intraperitoneal LPS injections, whereas IL-1Ra mRNA peaked after 3–6 h [41].

The study’s strengths include a well-characterized patient cohort not under drug treatment that potentially could affect the analysis and the use of CSF for analysis instead of peripheral blood.

These results need to be validated in future studies, and especially it would be interesting to further explore the role for Hrct1 signaling of fatigue and also to apply more sensitive and accurate assays for IL-1β measures in CSF.

## Conclusion

The main findings of this study indicate a functional network in which several IL-1β-related molecules in CSF influence fatigue in addition to the clinical factors of depression and pain. The neuropeptide Hcrt1 seems to participate in fatigue signaling, but probably not through the IL-1 pathway.

## Additional files

Additional file 1: **Figure S1.** The visual analog scale (fVAS) used for scoring of fatigue. (DOC 25 kb)

Additional file 2: **Figure S2.** a) Scree-plot of the eigenvalues of components 1–5 from PCA model with biochemical variables only. Components 1 and 2 had eigenvalues > 1 and were retained in the analysis. b) Scree-plot of the eigenvalues of components 1–6 from the PCA model with biochemical variables and fatigue (fVAS). The first three components had eigenvalues > 1 and were retained in the analysis. (TIFF 533 kb)

## Abbreviations

Hcrt1: Hypocretin-1
IL1-Ra: Interleukin-1 receptor antagonist
IL1RII: Interleukin-1 receptor type II
IL-1β: Interleukin-1 beta
IL-6: Interleukin-6
S100B: S100 calcium-binding protein B
