# Supplementary material for: Interleukin-1-related activity and hypocretin-1 in cerebrospinal fluid contribute to fatigue in primary Sjögren’s syndrome
Source: J Neuroinflammation. 2019 May 17;16:102. doi: 10.1186/s12974-019-1502-8 (PMC6525358; doi:10.1186/s12974-019-1502-8)
Supplement: Supplementary file 1 — Figure S1. The visual analog scale (fVAS) used for scoring of fatigue. (DOC 25 kb) [file 12974_2019_1502_MOESM1_ESM.doc]

Figure S1

# Fatigue Visual Analogue Scale

This questionnaire asks about how much of a problem fatigue has been for you in the last week.

To the left is “no fatigue” and to right “fatigue as bad as it can be”. Rate your fatigue by placing a marker at the point along the line that best represents your perception of fatigue over the last week.

Fatigue as bad

as it can be

No fatigue
