# Supplementary figures and images for: Interleukin-1-related activity and hypocretin-1 in cerebrospinal fluid contribute to fatigue in primary Sjögren’s syndrome
Source: J Neuroinflammation. 2019 May 17;16:102. doi: 10.1186/s12974-019-1502-8 (PMC6525358; doi:10.1186/s12974-019-1502-8)

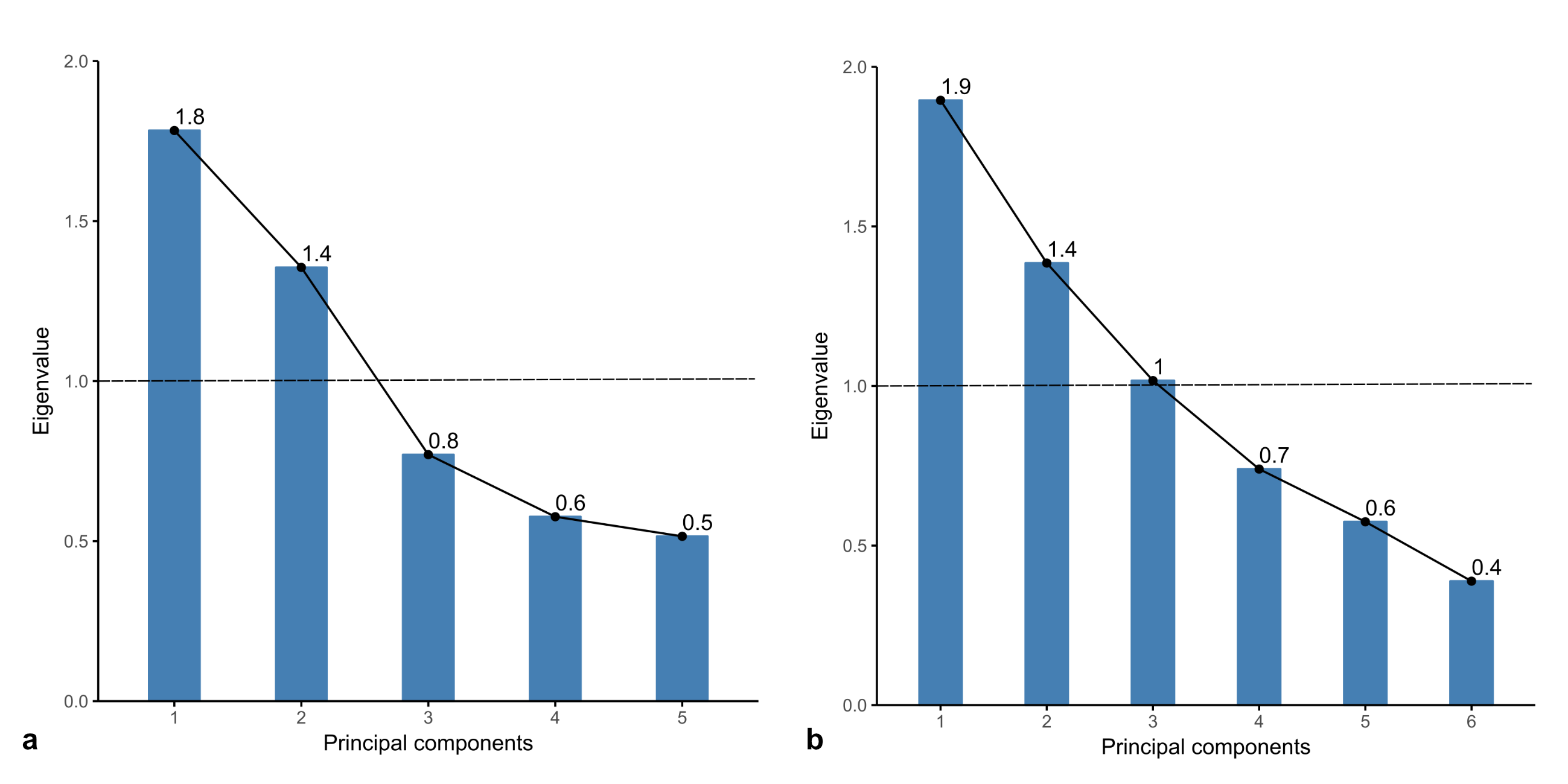

Supplement: Supplementary file 2 — Figure S2. a) Scree-plot of the eigenvalues of components 1–5 from PCA model with biochemical variables only. Components 1 and 2 had eigenvalues > 1 and were retained in the analysis. b) Scree-plot of the eigenvalues of components 1–6 from the PCA model with biochemical variables and fatigue (fVAS). The first three components had eigenvalues > 1 and were retained in the analysis. (TIFF 533 kb) [file 12974_2019_1502_MOESM2_ESM.tiff]
